# The Practical Physician

**Published:** 1890-02

**Authors:** 


### The Practical Physician.

At the Congress of American Physicians and Surgeons, held in Washington last September, Dr. William H. Draper, in his presidential address before the Association of American Physicians, presented in a very graphic and striking manner what should be regarded as the qualifications of the practical physician to-day. He is not a bacteriologist; he is not a pathologist; he is not a chemist or a physicist; he is not merely a therapeutist; he is not a specialist of any sort, nor does he look at clinical medicine from any limited horizon; but he is a man who, in some sense, is master of all these several branches of medical education by reason of combining as much as is possible of the sciences which these diffierent divisions represent, and thus perfects the most beneficent of all the arts. "It is he who, in his high position as the servant of humanity, must attain that wisdom which results from combining knowledge with the instinct and the skill for its useful application."—*Formulary and Drug. Magazine.*

